# Normofractionated irradiation and not temozolomide modulates the immunogenic and oncogenic phenotype of human glioblastoma cell lines

**DOI:** 10.1007/s00066-022-02028-8

**Published:** 2022-12-08

**Authors:** Julia Schatz, Alexandra Ladinig, Rainer Fietkau, Florian Putz, Udo S. Gaipl, Benjamin Frey, Anja Derer

**Affiliations:** 1 Translational Radiobiology, Department of Radiation Oncology, Universitätsklinikum Erlangen, Friedrich-Alexander-Universität Erlangen-Nürnberg (FAU), Universitätsstr. 27, 91054 Erlangen, Germany; 2 Department of Radiation Oncology, Universitätsklinikum Erlangen, Friedrich-Alexander-Universität Erlangen-Nürnberg (FAU), Erlangen, Germany; 3 Comprehensive Cancer Center Erlangen-EMN, Erlangen, Germany

**Keywords:** PD-L1, Clonogenicity, Glioblastoma cell immune phenotype, Immune checkpoint molecules, Epidermal growth factor receptor

## Abstract

**Purpose:**

Glioblastoma multiforme (GBM) is the most aggressive primary brain tumor, with an overall poor prognosis after diagnosis. Conventional treatment includes resection, chemotherapy with temozolomide (TMZ), and concomitant radiotherapy (RT). The recent success of immunotherapy approaches in other tumor entities, particularly with immune checkpoint inhibitors, could not be clinically transferred to GBM treatment so far. Therefore, preclinical analyses of the expression of both immune-suppressive and immune-stimulatory checkpoint molecules following treatment of human glioblastoma cells with RT and/or temozolomide is needed to design feasible radio(chemo)immunotherapy trials for GBM in the future.

**Methods:**

Five human glioblastoma cell lines (H4, HROG-06, U118, U138, U251) were analyzed regarding their clonogenic survival and cell death forms after chemotherapy (CT) with TMZ and/or normofractionated RT (5 × 2 Gy) via multicolor flow cytometry. Further, the tumor cell surface expression of immune-activating (OX40L, CD137L, CD70, and ICOSL) and immune-suppressive (PD-L1, PD-L2, HVEM) checkpoint molecules and of an oncogenic molecule (EGFR) were measured via multicolor flow cytometry after CT and RT alone or after RCT.

**Results:**

Normofractionated RT and not TMZ was the trigger of induction of predominantly necrosis in the glioblastoma cells. Notably, clonogenicity did not correlate with cell death induction by RT. The basal expression level of immune-suppressive PD-L1, PD-L2, and HVEM varied in the analyzed glioblastoma cells. RT, but not TMZ, resulted in a significant upregulation of PD-L1 and PD-L2 in all tumor cells investigated. Also, the expression of HVEM was increased after RT in most of the GBM cell lines. In contrast, normofractionated RT individually modulated expression of the stimulating immune checkpoint molecules CD70, CD137L, OX40L, and ICOSL1. The oncogenic factor EGFR was significantly increased by irradiation in all examined cell lines, albeit to a different extent. None of the investigated molecules were downregulated after the treatments.

**Conclusion:**

Normofractionated radiotherapy modulates the immunogenic as well as the oncogenic phenotype of glioblastoma cells, partly individually. Therefore, not only PD-L1 and PD-L2, but also other immunogenic molecules expressed on the surface of glioblastoma cells could serve as targets for immune checkpoint blockade in combination with RT in the future.

**Supplementary Information:**

The online version of this article (10.1007/s00066-022-02028-8) contains supplementary material, which is available to authorized users.

## Introduction

About 80% of malignant tumors of the central nervous system (CNS) are malignant gliomas and mainly incurable. Glioblastoma multiforme (GBM) is, with 16% of all primary and 54% of all malignant CNS neoplasms, the most common primary brain tumor in adults [1]. The average incidence rate is approximately 3 in 100,000 and the median age of diagnosis is 64 years [2]. GBM is the most aggressive glioma of astrocytic lineage, corresponding to grade IV based on the World Health Organization (WHO) classification. Its diffuse infiltrative and invasive growth makes complete resection practically impossible; therefore, radiation therapy (RT) combined with chemotherapy (CT) is the first-line treatment after maximal safe surgical resection [3]. The standard treatment with postoperative RT for GBM nowadays contains a dose of 1.8–2 Gy on 5 days per week for 6 weeks [4]. Temozolomide (TMZ), an oral alkylating chemotherapy agent, is typically given at a dose of 75 mg/m^2^ daily for 6 weeks, followed by a period of 1 month, before being restarted with 200 mg/m^2^ for 5 consecutive days per month continued for 6 months after RT [4]. Chemotherapeutic agents and RT act directly on the tumor cells by inducing DNA damage, cell cycle arrest, and immunogenic cancer cell death.

The latter, being particularly induced by fractionated RT with higher single doses, is mainly characterized by the release of immune-activating danger signals. These signals promote uptake of tumor peptides derived from irradiated cells by dendritic cells (DC), subsequently activating T cells by presenting those peptides.

Still, despite maximal possible resection and multimodal standard therapy, about 70% of GBM patients will experience disease recurrence and tumor progression within 1 year after diagnosis [5]. Not only the aggressive growth, but above all the extensive molecular heterogeneity of GBM as well as the paucity of GBM-infiltrating T‑cells [6] may contribute to the poor prognosis with a median survival of 15 months (3 months if untreated) [7] and less than 5% 5‑year survival rate after diagnosis. Due to the poor prognosis of GBM, immunotherapy has emerged as a promising approach, since it can be effective against many cancer entities even in primary situations [8, 9]. Thereby, a huge variety of immune-suppressive mechanisms in GBM represent the major barrier to immunotherapy, but a contribution of immune factors to therapeutic success remains very likely [10]. Tumor cells prevent their detection and eradication by immune cells through cell–cell contacts, soluble factors, and immune-suppressive molecules on the outer cell membrane [11].

Multiple coreceptors on the T cell surface, so called immune checkpoints, modulate immune responses and can bind to immune checkpoint molecules (ICM) on tumor cells. Physiologically, such immune checkpoint molecules, as regulators for immune proliferation and activation, play a key role in preserving immune homeostasis and self-tolerance and thus in preventing autoimmunity [12]. ICM effects can be immune suppressive as well as immune stimulatory. In recognition of this fact, several ICM inhibitor antibodies are currently being tested or have already been approved for a number of cancer entities; the so-called immune checkpoint inhibitors (ICI). For example, agents targeting programmed cell death protein 1 (PD-1), its ligand PD-L1, or cytotoxic T lymphocyte-associated antigen 4 (CTLA4) receptors have been shown to have antitumor activity in different tumor entities such as melanoma, non-small cell lung cancer, and renal cancer [13]. However, monotherapy with ICI, such as anti-PD1 therapy as that most studied, showed only limited success in GBM [6, 14]. Therefore, one step towards an effective immunotherapy against GBM is to gain understanding of the genetic and phenotypic heterogeneity of GBM as well as its microenvironmental communication and different forms of immune suppression. In the following, we shortly introduce the ICMs we focused on in our analyses.

A key ICM with immune-suppressive character is programmed cell death receptor 1 (PD-1) and its ligands programmed death ligand 1 (PD-L1, CD274, B7-H1) and PD-L2 (CD273, B7-DC) that belong to the B7/CD28 superfamily [15]. PD‑1 is expressed by activated T cells but not restricted to them, as it can be found on natural killer cells (NKs) [16], B cells [17], and dendritic cells (DCs) [18] as well. Under healthy physiological conditions, binding of PD-L1 and PD-L2 to their receptor PD‑1 functions as a negative feedback regulation to suppress stimulated T cells, in order to prevent an overshooting T cell reaction and thus maintain leukocyte homeostasis within the body. However, tumor cells take advantage of this immune-suppressive mechanism by expressing PD-L1 and PD-L2 themselves [19]. In addition, RT further increases PD-L1 expression on tumor cells [20].

Another immune checkpoint molecule expressed by glioma cells is the herpes virus entry mediator (HVEM; CD270), which is a member of the tumor necrosis factor (TNF) receptor superfamily (TNFRSF14) and found to be expressed on hematopoietic as well as non-hematopoietic cells. HVEM binds to molecules of the immunoglobulin (Ig) superfamily (BTLA, CD272) as well as TNF proteins (LIGHT, TNFSF14, or CD258), both of which are expressed on a variety of immune cells such as T cells, B cells, DCs, and others. Simultaneously, HVEM acts as a signaling receptor as well as a ligand for inhibitory receptors. Thus, HVEM can act costimulatory or inhibitory on T cells depending on binding partners and bidirectional signaling capacity. In cancer, the binding of HVEM activates nuclear factor k-light-chain-enhancer of activated B cells (NFkB) and phosphatidylinositol 3-kinase (PI3K/Akt) signaling, which can lead to proliferation and survival signaling. In addition, HVEM was found to be elevated in aggressive gliomas [21].

Besides immune-suppressive ICMs, those with immune-activating properties do exist: OX40L is, like its ligand OX40 (CD134), a member of the TNF/TNFR family and found to be expressed on antigen presenting cells (APCs) (BCs, DCs, and macrophages) [22] as well as other lymphoid and nonlymphoid cells. It functions as a costimulatory molecule in conjunction with T cell receptor engagement and affects T cell proliferation and survival [22]. Shibahara et al. found OX40L mRNA to be expressed in glioblastoma specimens and higher expression levels were associated with prolonged progression-free survival [23]. Moreover, in a murine glioblastoma model, they found that OX40L expression modulated the adaptive immunity in dependence on the microenvironment [23]. CD137L (4-1BBL, Tnfsr9-l) is a member of the TNF receptor–TNF ligand family and mostly expressed on T cells and APCs [24]. The soluble form of 4‑1BB was reported in RA patients and 4‑1BB expression increases in PBMCs after γ‑irradiation. Professional APCs, such as DCs and macrophages, express their ligand after stimulation, and binding of 4‑1BBL to its receptor leads to CD4+ and CD8+ T cell activation. Salih et al. demonstrated that 4‑1BBL is also present on some carcinoma cells of solid tumors [25]. Immunohistochemical analyzes showed 4‑1BBL-positive cells in 26.3% of glioblastoma tissue samples [26]; however, the expression was not correlated with overall survival of GBM patients. Activated DCs, B cells, conventional, and regulatory T cells and natural killer cells express CD70 (CD27L, TNFSF7) after immune activation. In addition, epithelial cells can acquire CD70 expression upon malignant transformation. Its ligand, CD27, is expressed by the lymphoid lineage on T cells, B cells, and natural killer cells. CD70 has a costimulatory function by promoting TCR/CD3-induced proliferation of naïve CD4+ and CD8+ T cells and the generation of effector cells. CD70 was found to be overexpressed on isocitrate dehydrogenase (IDH) primary and low-grade gliomas as well as GBMs and associated with poor survival in the investigated subgroups [27]; further, CD70-specific CAR T cells showed an antitumor response against CD70+ gliomas in xenograft and syngeneic glioma models [27].

The inducible costimulatory ligand (ICOSL) belongs to the B7 family of costimulatory molecules and regulates CD4 and CD8 T cell responses via interaction with ICOS on activated T cells. ICOSL was found to be expressed by 7 of 12 glioma cell lines and neutralizing ICOSL antibodies reduced Th1 and Th2 cytokine levels in cocultures of PBMCs or T cell subsets with glioma cells [28].

In addition to ICM, we also included the epidermal growth factor receptor (EGFR) in our analyses as one key oncogenic factor. Dysregulated HER1/EGFR is found in 40% to 50% of glioblastoma [29]. Amplification of EGFR signaling can be dependent on ligand binding with activation of canonical signaling pathways. In addition, *EGFR* can harbor point mutations or deletions that lead to constitutive activation of the receptor and independence from ligand binding. Its permanent activity leads to resistance to apoptosis and/or intensified angiogenesis, proliferation, and invasion [30] as well as treatment resistance. Several agents such as tyrosine kinase (TK) inhibitors, antibodies, radioimmunoconjugates, ligand–toxin conjugates, or RNA-based agents have been developed to target HER1/EGFR or its mutant form, EGFRvIII. However, treatment strategies targeting EGFR have thus far failed in clinical trials [30].

Taken together, despite modern treatment schemes, patients still face a very poor prognosis after GBM diagnosis. Its location within the brain and the fact that the tumor itself is considered not to be immunogenic hampers research and treatment development and reduces therapeutic options. To date, the success of immunotherapeutic approaches in GBM, especially the use of ICI, is limited. Still, in various tumor entities the combination of radiotherapy and ICI is viewed to be more effective than either one alone, which was in a large part due to the immunomodulating impact of RT [31]. It might appear that, given the right circumstances, ICI could be a promising approach for combination therapy with conventional standard-of-care in GBM. Therefore, the choice of ICI needs to be adapted to the individual molecular pattern of tumor as well as the impact of the therapeutic process. Subsequently, the benefits of the immunogenic impact of RT could be used and the immune-suppressive mechanisms of the tumor cells could be considered for treatment intervention. However, to date, only little is known about how RT and CT affect immune checkpoint molecule expression in GBM. Thus, we need to gain knowledge regarding the expression pattern of potential immune checkpoint targets and the impact of conventional treatment on their expression. In this study, we hypothesized that GBM tumors differ in the basal expression of ICM and that conventional RT and TMZ modulates the expression of ICM. To test this, we investigated the expression of inhibitory and activating immune checkpoint molecules as well as EGFR as an oncogenic factor in five human glioblastoma cell lines at basal level and after treatment with fractionated RT and/or TMZ.

## Materials and methods

### Cell culture

The human glioblastoma cell lines HROG-06, U138, and U251 were obtained from Cell Line Service (CLS; Eppelheim, Germany), while H4 and U118 cells were obtained from ATCC (Manassas, VA, USA). The GBM cell line U251 was maintained in Dulbecco’s modified Eagle’s medium (DMEM; PAN-Biotech GmbH, Aidenbach, Germany); H4, U138, and U118 cells in DMEM supplemented with 4.5 g/l D‑glucose, L‑glutamine, and pyruvate (Life Technologies Limited, Paisley, UK); and HROG-06 cells in DMEM/F-12 nutrient mixture (1:1) supplemented with GlutaMAX (Life Technologies Limited, Paisley, UK). Each medium was supplemented with 10% fetal bovine serum (FBS; Biochrom AG, Berlin, Germany) and 1% penicillin-streptomycin (100 U/ml penicillin and 100 μg/ml streptomycin; Invitrogen, Darmstadt, Germany). Cells were grown in cell culture flasks (Cellstar; Greiner BioOne, Nürtlingen, Germany) at 37 °C in humidified air with 5% CO_2_. Temozolomide (TMZ; Sigma-Aldrich, Munich, Germany) was dissolved at a stock concentration of 100 mM in dimethyl sulfoxide (DMSO; Sigma-Aldrich, Munich, Germany) and stored at −20 °C. TMZ was diluted in culture medium immediately before treatment of cells to a concentration of 20 µM.

### Clonogenic survival assays

The glioblastoma cell lines were harvested and plated in petri dishes at appropriate dilutions adapted for each cell line and treatment. When cells were attached to the dishes, they were left untreated (control) or irradiated with one single dose of either 1, 2, 4, 6, 8, or 10 Gy with an X‑ray generator (GE Inspection Technologies, Hürth, Germany) and placed in an incubator at 37 °C in humidified air with 5% CO_2_. Cells were incubated until the control dishes had formed sufficiently large colonies, which were considered representative when they contained at least 50 cells. Then medium was removed, the colonies were washed with PBS, fixed, and stained using 80% ethanol and 8^0^/_00_ methylene blue (Sigma Aldrich, Munich, Germany). Cell colonies were counted and plating efficiency-based calculation of survival fractions was performed. In brief, plating efficiency was calculated by dividing the counted number of colonies by the number of seeded cells in the untreated control dishes. The surviving fraction of cells in each condition is normalized by the plating efficiency of the unirradiated control cells. For all cell lines, each experiment was done in triplicate and three to five independent experiments were performed, depending on the cell line used (H4, *n* = 3; HROG-06, *n* = 5; U118, *n* = 4; U138, *n* = 5; U251, *n* = 3).

### Treatment with TMZ and the irradiation procedure

The glioblastoma cell lines were seeded in 75 cm^2^ flasks at different densities between 1 and 6 × 10^5^ cells, depending on treatment and cell line. Cells were irradiated with a clinically relevant total weekly dose of 10 Gy, with a single dose of 2 Gy (representing a daily dose in GBM standard therapy) on 5 consecutive days, started 24 h after seeding with an X‑ray generator (GE Inspection Technologies, Hürth, Germany).

TMZ was added to the respective flasks directly after irradiation. The cells were harvested 24 h after the last irradiation and stained for cell death detection as well as immune checkpoint expression via flow cytometry.

### Cell death detection

Induction of cell death forms by the respective treatments were examined using annexin A5 (AnxA5)-FITC/propidium Iodide (PI) staining. Treated cells were seeded into 96-well plates at a density of 1 × 10^6^ cells/ml cell suspension, thus 1.5 × 10^5^ cells per well, and incubated for 30 min at 4 °C in the dark with 0.5 μg/ml AnxA5-FITC (ThermoFisher/Geneart, Regensburg, Germany; FluoroTaq FITC conjugation Kit, Sigma-Aldrich, Munich, Germany) and 1 μg/ml PI (Sigma-Aldrich, Munich, Germany) in Ringer’s solution (Fresenius, Kabi, France). Samples were measured by flow cytometry (CytoFLEX S, Beckman Coulter, Brea, USA) and analyzed by the associated Kaluza 2.1 Software®. Viable cells show neither binding of AnxA5 nor staining with PI. Early apoptotic cells are positive for AnxA5 but negative for PI staining, whereas late apoptotic and necrotic cells are positive for both AnxA5 and PI.

### Detection of immunogenic and oncogenic factors

For surface staining of immunogenic and oncogenic factors, cells were transferred to 96-well plates at a density of 1.5 × 10^5^ cells per well and incubated with saturated fluorochrome-labeled antibodies in FACS buffer (2 mM EDTA and 2% FCS in PBS). The antibodies used were PD-L1 (anti-human CD274, Brilliant Violet 605, BioLegend, San Diego, CA, USA), PD-L2 (anti-human CD273, APC, BioLegend), ICOSL (mouse anti-human CD275, BV421, BioLegend), HVEM (anti-human CD270, APC, BioLegend), Ox40L (anti-human CD252, PE, BioLegend), CD137L (anti-human CD137L, Brilliant Violet 421, BioLegend), and EGFR (anti-human EGFR, PE, BioLegend). A zombie NIR dye (BioLegend) was used to assess vital cells. In addition, irradiation of cells changes their structure and thereby enhances their autofluorescence in various fluorescence channels, which is why from each condition, one sample was additionally stained with the vitality zombie NIR dye alone and analyzed separately to the stained samples (Supplementary Fig. 2). Surface expressions were analyzed by flow cytometry and the associated Kaluza 2.1 Software®. The gating strategy of the live cells and the staining panels are depicted in Supplementary Fig. 1.

### Statistical analyses

Statistical analyses were done using GraphPad Prism software (GraphPad software, Inc., San Diego, CA, USA). All data are presented as mean ±SEM. Statistical significance was calculated as stated in the figure description. Significances are indicated as follows: *p < 0.05, **p < 0.01, and ***p < 0.001.

## Results

### Irradiation and not chemotherapy is the key trigger of cell death induction in glioblastoma cells

**Fig. 1:**
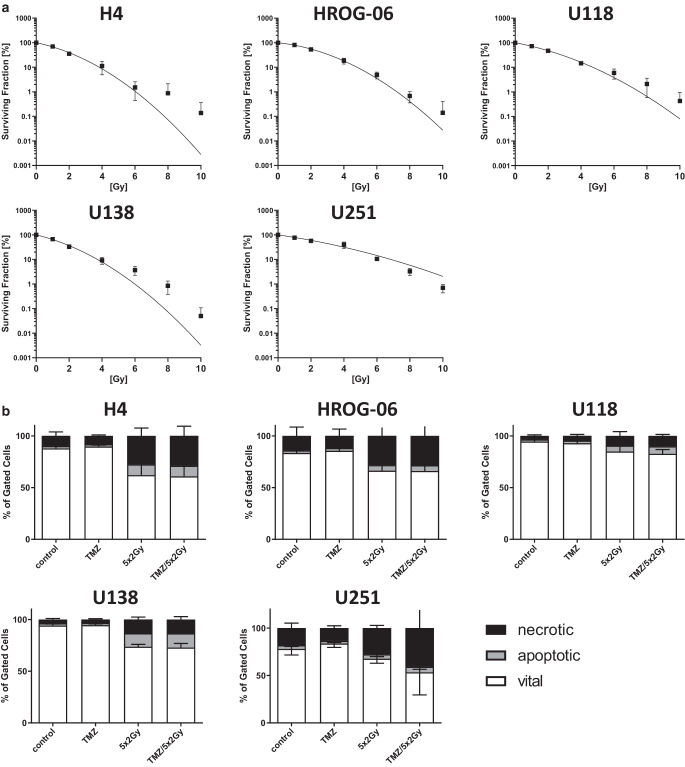
Clonogenic survival and cell death forms of glioblastoma cell lines after irradiation. Five human glioblastoma cell lines (H4, HROG-06, U118, U138, U251) were analyzed for their clonogenic survival as well as cell death forms after irradiation. **a** Analyses for clonogenic survival were performed after irradiation with a single dose of 2, 4, 6, 8, or 10 Gy radiotherapy (RT). Joint data of *n* independent experiments (H4, *n* = 3; HROG-06, *n* = 5; U118, *n* = 4; U138, *n* = 5; U251, *n* = 3) are presented as mean ± SEM. **b** For determination of cell death forms, the glioblastoma cell lines were analyzed 24 h after single or multimodal treatment with either chemotherapeutic agent (temozolomide, *TMZ*), fractionated radiotherapy (RT; 5 × 2 Gy), or combination radiochemotherapy. Vital cells (*white*) are defined as annexin 5A (AnxA5)–/PI–, apoptotic cells (*grey*) as AnxA5–/PI+, and necrotic cells (*black*) as AnxA5+/PI+. Joint data of *n* independent experiments (H4, *n* = 4; HROG-06, *n* = 3; U118, *n* = 4; U138, *n* = 4; U251, *n* = 3) are presented as mean ± SEM

First, we determined the radiosensitivity of the human glioblastoma cell lines H4, HROG-06, U118, U138, and U251 by performing colony formation assays. The cell lines were exposed to various doses of radiation (0 to 10 Gy) and formed colonies (>50 cells) were counted. Irradiation significantly reduced the colony formation of all cell lines, but to a different extent (Fig. 1a). U251 cells were the most radioresistant cells in this analysis. Next, we evaluated the impact of conventional glioblastoma treatment with normofractionated irradiation (5 × 2 Gy) and TMZ on the induction of tumor cell death. Fig. 1b displays cell death forms of H4, HROG-06, U118, U138, and U251 glioblastoma cells 24 h after the last treatment with fractionated RT and/or TMZ. In all cell lines, fractionated RT resulted in an increased number of both apoptotic and necrotic cells. In cell lines H4, HROG-06, and U251 the number of necrotic cells was higher than that of apoptotic cells, while in cell lines U138 and U118, fractionated RT induced a smaller proportion of apoptotic and necrotic cells. Thus, a radiosensitizing effect regarding apoptosis and necrosis induction by TMZ could not be observed (Fig. 1b)—neither alone nor in combination with RT. Nevertheless, more than 50% of all cells were still viable 24 h after treatment. Of note is that while U251 cells were radioresistant regarding clonogenicity, they were sensitive to necrosis induction following RCT.

### Fractionated RT induces expression of inhibitory immune checkpoint molecules PD-L1 and PD-L2 on glioblastoma cells

**Fig. 2:**
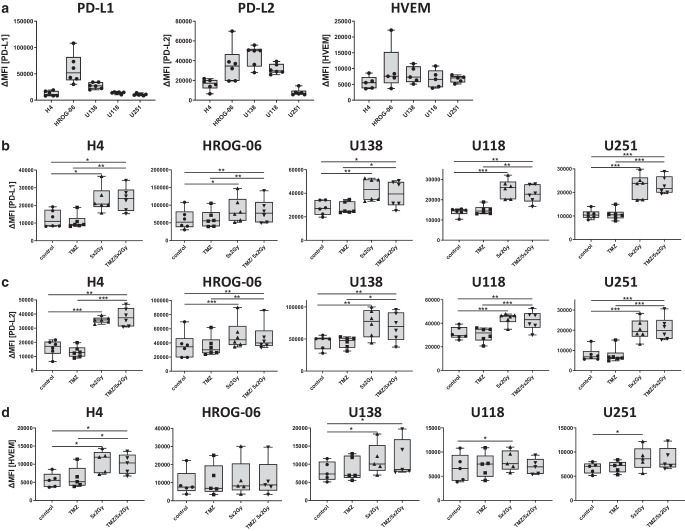
Expression of inhibitory immune checkpoint molecules after irradiation and (temozolomide, *TMZ*). Five human glioblastoma cell lines (H4, HROG-06, U118, U138, U251) were analyzed for cell surface expression of (**a** and **b**) PD-L1, (**a** and **c**) PD-L2, and (**a** and **d**) HVEM 24 h after single or multimodal treatment with either chemotherapeutic agent TMZ, fractionated radiotherapy (RT; 5 × 2 Gy), or combination radiochemotherapy for 5 consecutive days. Fig. 2a shows the basal expression of PD-L1, PD-L2, and HVEM in comparison between the cell lines. Expression of the indicated molecule was determined on vital cells by staining with the respective antibody and consecutive analysis by multicolor flow cytometry. Joint data of six (PD-L1 and PD-L2) or five (HVEM) independent experiments are presented as mean ± SEM and analyzed by two-tailed t‑test as calculated via Graph Pad Prism (GraphPad software, Inc.). *PD-L1/2* Programmed death ligand 1 and 2, *HVEM* Herpes virus entry mediator. *p < 0.05; **p < 0.01; ***p < 0.001

While cell death analyses give hints about the basic immune properties of tumor cells in the initiation phase of an immune response following treatment with RT and/or CT [32], analyses of surface expression of immune checkpoint molecules provide information about the immune phenotype of the tumor cells that particularly affects the effector phase of an anti-tumor immune response [33]. Therefore, we treated different glioblastoma cell lines (H4, HROG-06, U138, U118, U251) with normofractionated irradiation of 2 Gy on 5 consecutive days with or without TMZ treatment. The following day, surface expression of immune checkpoint molecules was analyzed via flow cytometry. The first new observation was that the basal expression level of the examined immune suppressive checkpoint molecules PD-L1, PD-L2, and HVEM is different in glioblastoma cells, with the highest levels of expression of PD-L1 and PD-L2 on HROG-06 cells (Fig. 2a). Of note, all examined glioblastoma cell lines express the mentioned immune checkpoint molecules on their cell surface (Fig. 2a–d). Fractionated irradiation and additional treatment with TMZ of tumor cells significantly increased the expression of the inhibitory checkpoint molecules PDL‑1 and PDL‑2 (Fig. 2b and c). The expression of HVEM was significantly increased by RT in all cell lines, except for HROG-06 (Fig. 2d). However, TMZ (alone or combined with RT) had no significant influence on the expression of inhibitory checkpoint molecules, neither inhibitory nor enhancing.

### Fractionated RT individually modulates expression of the stimulating immune checkpoint molecules CD70, CD137L, OX40L, and ICOSL1 on glioblastoma cells

**Fig. 3:**
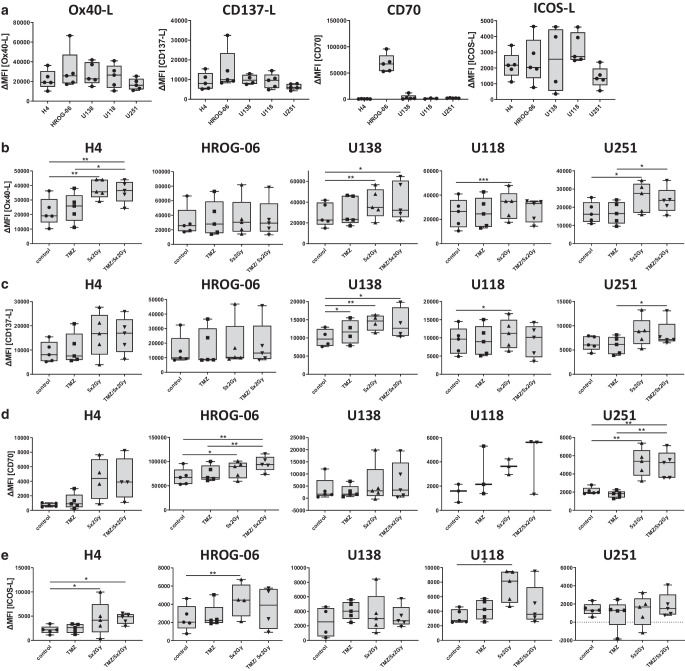
Expression of activatory immune checkpoint molecules after irradiation and temozolomide (*TMZ*). Five human glioblastoma cell lines (H4, HROG-06, U118, U138, U251) were analyzed for cell surface expression of (**a** and **b**) OX40L, (**a** and **c**) CD137L, (**a** and **d**) CD70, and (**a** and **e**) ICOSL 24 h after single or multimodal treatment with either chemotherapeutic agent TMZ, fractionated radiotherapy (RT; 5 × 2 Gy), or combination radiochemotherapy for 5 consecutive days. **a** Basal expression of OX40L, CD137L, CD70, and ICSOL in comparison between the cell lines. Expression of the indicated molecule was determined on vital cells by staining with the respective antibody and consecutive analysis by multicolor flow cytometry. Joint data of five independent experiments are presented as mean ± SEM and analyzed by two-tailed t‑test as calculated via Graph Pad Prism (GraphPad software, Inc.). *ICOSL* Inducible T‑cell Co-stimulator Ligand. *p < 0.05; **p < 0.01; ***p < 0.001

Next, we investigated the cell surface expression of the activating immune checkpoint molecules OX40L, CD137L, CD70, and ICOSL. Of note, all analyzed immune checkpoint molecules were found to be expressed on all examined glioblastoma cell lines H4, HROG-06, U138, U118, and U251 (Fig. 3a–e). OX40L was upregulated by RT alone or combined with TMZ in four (H4, U138, U118, and U251) out of five glioblastoma cell lines (Fig. 3b). In contrast, the expression of CD137L was significantly upregulated only on U138, U118, and U251 following RT or RCT (Fig. 3c). The expression of CD70 is not influenced by TMZ treatment or RT in H4, U138, or U118, but is significantly upregulated by RT with and without TMZ in cell lines HROG-06 and U251 (Fig. 3d). The basal expression level of ICOSL is similar in all cell lines. RT significantly induced the expression of ICOSL on H4, HROG-06, and U118 cells (Fig. 3e).

### Expression of EGFR as an oncogenic factor is increased by RT in glioblastoma cells

**Fig. 4:**
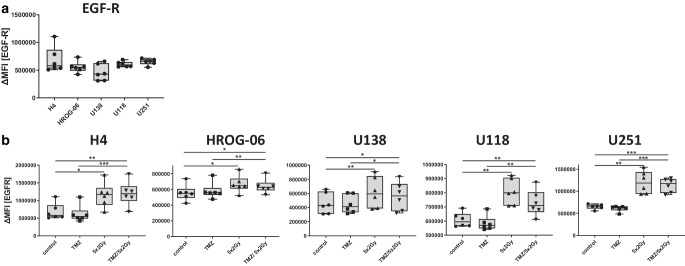
Expression of EGFR molecule expression after Irradiation and temozolomide (*TMZ*). Five human glioblastoma cell lines (H4, HROG-06, U118, U138, U251) were analyzed for cell surface expression of EGFR 24 h after single or multimodal treatment with either chemotherapeutic agent TMZ, fractionated radiotherapy (RT; 5 × 2 Gy), or combination radiochemotherapy for 5 consecutive days (**b**). **a** shows the basal expression of EGFR comparison between the cell lines. Expression of the indicated molecule was determined on vital cells by staining with the respective antibody and consecutive analysis by multicolor flow cytometry. Joint data of six independent experiments are presented as mean ± SEM and analyzed by two-tailed t‑test as calculated via Graph Pad Prism (GraphPad software, Inc.). *EGF‑R* Epidermal Growth Factor Receptor. *p < 0.05; **p < 0.01; ***p < 0.001

The expression of EGFR was significantly increased by irradiation in all examined cell lines, albeit to differing extents. A strong induction of expression after RT could be detected in H4, U118, and U251 glioblastoma cells. HROG-06 and U138 showed a significant upregulation after RT, although the expression level is not doubled. Again, there is no additive effect of TMZ administration (Fig. 4a and b).

## Discussion

Despite modern radiotherapy concepts and adjuvant chemotherapeutic treatment schemes, glioblastoma patients are still confronted with a very poor prognosis, which is why novel multimodal therapy schemes are urgently needed. One of the latest therapeutic options is involvement of immune checkpoint inhibitors in the conventional treatment setting of radio(chemo)therapy. However, so far, the success of immunotherapy concepts in first clinical trials in glioblastoma seems limited, whereby many studies are ongoing and results are still pending [6, 14].

Today, several general mechanisms regarding glioma treatment resistance have become clear, such as radio- and chemoresistance of the tumor cells [34], secretion of immune-suppressive cytokines, and the plasticity and heterogenicity of glioblastoma cells [35], all of which helps the tumor to escape immune surveillance.

Still, one reason for a limited success of immune therapy concepts might be that not much is known regarding the expression and regulation of immune checkpoint molecules within the tumor in terms of spatial and temporal aspects. It is important to consider that these receptor–ligand pairs are able to successfully suppress immune responses of, e.g., cytotoxic T cells, a mechanism that is highly important under normal physiological conditions but also blocks anti-tumor responses. Thus, we analyzed the basal expression of several immune checkpoint molecules as well as the impact of the conventional treatment scheme of normofractioned radiotherapy (5 × 2Gy) and temozolomide administration.

We first screened five human glioblastoma cell lines (H4, HROG-06, U118, U138, U251) for their treatment response regarding colony formation and cell death. Normofractionated irradiation effectively inhibited colony formation and induced apoptosis and necrosis in all investigated cell lines, while its combination with TMZ could not further potentiate these effects (Fig. 1a, b). Of note, the proportion of vital cells after the treatment remained relatively high in all cell lines, reflecting the radiation resistance of glioblastoma tumor cells.

Interestingly, the loss of colony formation is often not correlated with the number of dying cells. In addition, U118 and U138 show an enhanced radioresistance regarding cell death induction, which is supposed to be surviving driven [36] and can be detected more impressively when analyzing cell death rather than colony-forming capability. In this regard, induction of cell death is not correlated with colony-forming capability in the case of U118, U138, and U251 glioblastoma cells. However, it is known that glioblastoma cells are also capable of undergoing senescence or autophagy, also just under TMZ treatment [37]. As necrosis was the prominent cell death form, one could speculate that the glioblastoma cells have immunogenic potential, since necrotic cells are characterized by disturbed membrane integrity that goes along with the release of immune-activation danger signals that activate dendritic cells and thereby prime anti-tumor immune responses (summarized in [38]).

To get deeper insights into how the immune phenotype of glioblastoma cells might affect the effector phase of anti-tumor immune responses following standard tumor treatments, we analyzed for the first time in detail the expression of immune checkpoint molecules on the tumor cell surface. These molecules induce or preserve an immunosuppressive environment, defending against the attack of T cells, macrophages, and microglia [39].

Key immunosuppressive immune checkpoint molecules, PD-L1 or PD-L2, were significantly upregulated in all analyzed glioblastoma cell lines following RT. But it is not only important to note that RT alone has a major impact on this upregulation, but also that temozolomide does not have a role here. This has already been described for other model systems [20].

RT-induced upregulation of PD-L1 and PD-L2 can be exploited therapeutically by immune checkpoint inhibitors (ICI). The concept of “in situ vaccination” [40], characterized by, e.g., induction of immunogenic necrosis, could therefore also be applied to glioblastoma [41] when restoring anti-tumor responses by immune checkpoint inhibitors. The first hints of this have already been seen in clinical trials, where the combination therapy of RT and ICI performs better than ICI alone [42]. Since the expression of not only PD-L1 is increased but also that of PD-L2 in a similar manner, the inhibition of the PD‑1 receptor on T‑cells may have a stronger effect.

Another immunosuppressive ligand on tumor cells, the herpes virus entry mediator (HVEM), that has been shown to be expressed in the microenvironment of GBM, driving Treg induction and stronger immunosuppression, is correlated with a reduced outcome in patients if overexpressed in the GBM tissue [21]. Interestingly, RT- or RT plus TMZ-induced expression of HVEM did not show a similar pattern to PD-L1/2 expression and therefore seems to have a more personalized expression pattern in GBM.

The transmembrane glycoprotein CD 70, a member of the tumor necrosis factor (TNF) superfamily, is transiently expressed on activated T and B cells as well as mature dendritic cells and is the only known ligand for CD27, but is minimally expressed in most normal tissues [43]. Nevertheless, a stronger expression could be detected on recurrent glioblastoma, which is why this ligand should be used as a target for CAR T cell therapies [43]. Initial results are promising, but there seems to be an advantage in recurrent GBM compared to primary GBM. Our analyses indicate that basal expression on the cells is low, but that especially in U251 and HROG-06 cells, an increased expression was induced by RT, making this therapeutic approach suitable in a combinatory radioimmunotherapy in selected GBM patients.

The OX40 ligand as well as high levels of OX40L mRNA were detected in GBM tumor cells. Both are associated with prolonged progression-free survival in GBM patients [23]. Despite initial attempts to use this ligand for immunotherapy, the results were rather sobering and strongly dependent on the micromilieu [44]. This is probably because the basal expression is too low and attempts at combined therapy were never undertaken. Based on our in vitro data, this approach should be further considered, since all analyzed cells, except HROG cells, show a significant upregulation of OX40L after RT. The detection of ICOSL and CD137 revealed a heterogenous expression pattern, reflecting once more the heterogenicity of GBM, even when looking at immune checkpoint molecules [45, 46].

It is nowadays well established that abnormal expression of EGFR can regulate tumor cell proliferation, migration, differentiation, and homeostasis, and even in GBM, more than half of patients have genetic variants of EGFR. Inhibition of EGFR has therefore already been tested in clinical trials with varying degrees of success [47]. Therefore, it is known that blocking HER1/EGFR is not sufficient to induce a sufficient clinical benefit. Especially in the treatment of GBM, EGFR targeting (whether with small molecule inhibitors or monoclonal antibodies) has by far not shown the effect that had been hoped for [47, 48]. Therefore, a multiple-target approach, and especially a multimodality approach in combination with radio- and/or radiochemotherapy, could be a beneficial concept [29]. Our data show that in most of the cell lines studied, regulation of EGFR takes place when the cells were treated with RT or RT-TMZ. Interestingly, the increase in EGFR expression is greatest in U251 and U118 cells, which could be due to a mutation in *PTEN* [49].

**Fig. 5:**
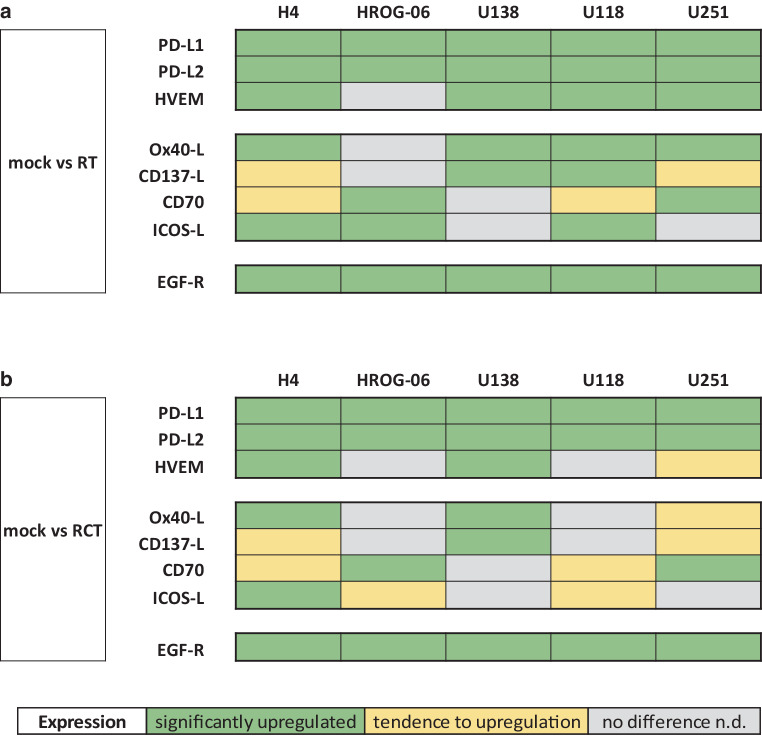
Overview of immunogenic and oncogenic molecule expression on glioblastoma cell lines after radio(chemo)therapy. The heat map summarizes the expression of immunogenic and oncogenic molecules on glioblastoma cell lines after radio(chemo)therapy. Human glioblastoma cell lines (H4, HROG-06, U118, U138, U251) were analyzed for cell surface expression of the indicated molecules 24 h after (**a**) fractionated radiotherapy (RT) alone (5 × 2 Gy) or (**b**) multimodal treatment with chemotherapeutic agent temozolomide (TMZ) plus fractionated radiotherapy (RCT; 5 × 2 Gy). Expression of the indicated molecule was determined on vital cells by staining with the respective antibody and consecutive analysis by multicolor flow cytometry. A *green box* defines a statistically significant upregulation of the respective molecule, a *yellow box* defines a tendency towards upregulation, and a *grey box* indicates no difference in expression after the respective treatment. *EGFR* Epidermal Growth Factor Receptor, *HVEM* Herpes virus entry mediator, *mock* mock-treated, *ICOSL* Inducible T‑cell Co-stimulator Ligand, *CD137‑L* ligand of CD137, *Ox40‑L* ligand of Ox40, *PD-L1/2* Programmed death ligand 1 and 2, *RT* radiotherapy, *RCT* radiochemotherapy

In summary, regulation of the expression of immune checkpoint molecules on the surface of GBM cells after RT in particular is heterogeneous and TMZ does not strongly affect it (Fig. 5). RT particularly increased the expression of PD-L1, PD-L2, HVEM, and EGFR. A significant difference between RT and RT-TMZ treatment could not be detected. Nevertheless, it should be noted that the majority of clinical trials that started to target the molecules we investigated were not very successful [39, 50, 51]. This may be due to the fact that the vast majority of studies were not designed with a multimodal treatment approach (including radiotherapy). On the basis of our in vitro data, expression of immune checkpoint molecules should be individually screened particularly following RT in patients with GBM. This information might contribute to adaptations of multimodal radioimmunotherapies also for GBM in the future.

### Supplementary Information

**Supplementary Fig. 1: Gating strategy for flow cytometric analyses. **Gating strategy of the immune checkpoint panels. **(A)** First cellular events were detected via FSC-A/SSC‑A. Next, singlets were identified by excluding duplets and cell aggregates (FSC-A/FSC-H) and pre-gated for viable cells (FSC/SSC, zombie) for identification of surface molecules **(B)** PD-L1, PD-L2, ICOS‑L, and EGF‑R (antibody panel 1) as well as **(C)** OX40‑L, CD137‑L, CD70, and HVEM (Antibody Panel 2).

**Supplementary Fig. 2:** **Exemplary histogram of PD-L1 surface expression of mock-treated and irradiated U118 and U251 Cells.** **(A)** U118 and **(B)** U251 cells were analyzed for cell surface expression of PD-L1 24 h after mock treatment or fractionated radiotherapy (RT) (5 × 2 Gy) for 5 consecutive days. Each experimental sample was analyzed in two test samples via flow cytometry, one with the stained panel (all antibodies plus the cell dye zombie included) and the other one unstained (only cell dye zombie included), to take the autofluorescence of the treated cells into account. Exemplary histograms show PD-L1 surface expression of U118 **(A)** and U251 **(B)** cells after mock treatment and irradiation.
