# Supplementary material for: Normofractionated irradiation and not temozolomide modulates the immunogenic and oncogenic phenotype of human glioblastoma cell lines
Source: Strahlenther Onkol. 2022 Dec 8;199(12):1140–51. doi: 10.1007/s00066-022-02028-8 (PMC10673751; doi:10.1007/s00066-022-02028-8)
Supplement: Supplementary file 1 — Supplementary Fig. 1: Gating strategy for flow cytometric analyses. Gating strategy of the immune checkpoint panels. (A) First cellular events were detected via FSC-A/SSC‑A. Next, singlets were identified by excluding duplets and cell aggregates (FSC-A/FSC-H) and pre-gated for viable cells (FSC/SSC, zombie) for identification of surface molecules (B) PD-L1, PD-L2, ICOS‑L, and EGF‑R (antibody panel 1) as well as (C) OX40‑L, CD137‑L, CD70, and HVEM (Antibody Panel 2). [file 66_2022_2028_MOESM1_ESM.pptx]

## Slide 1
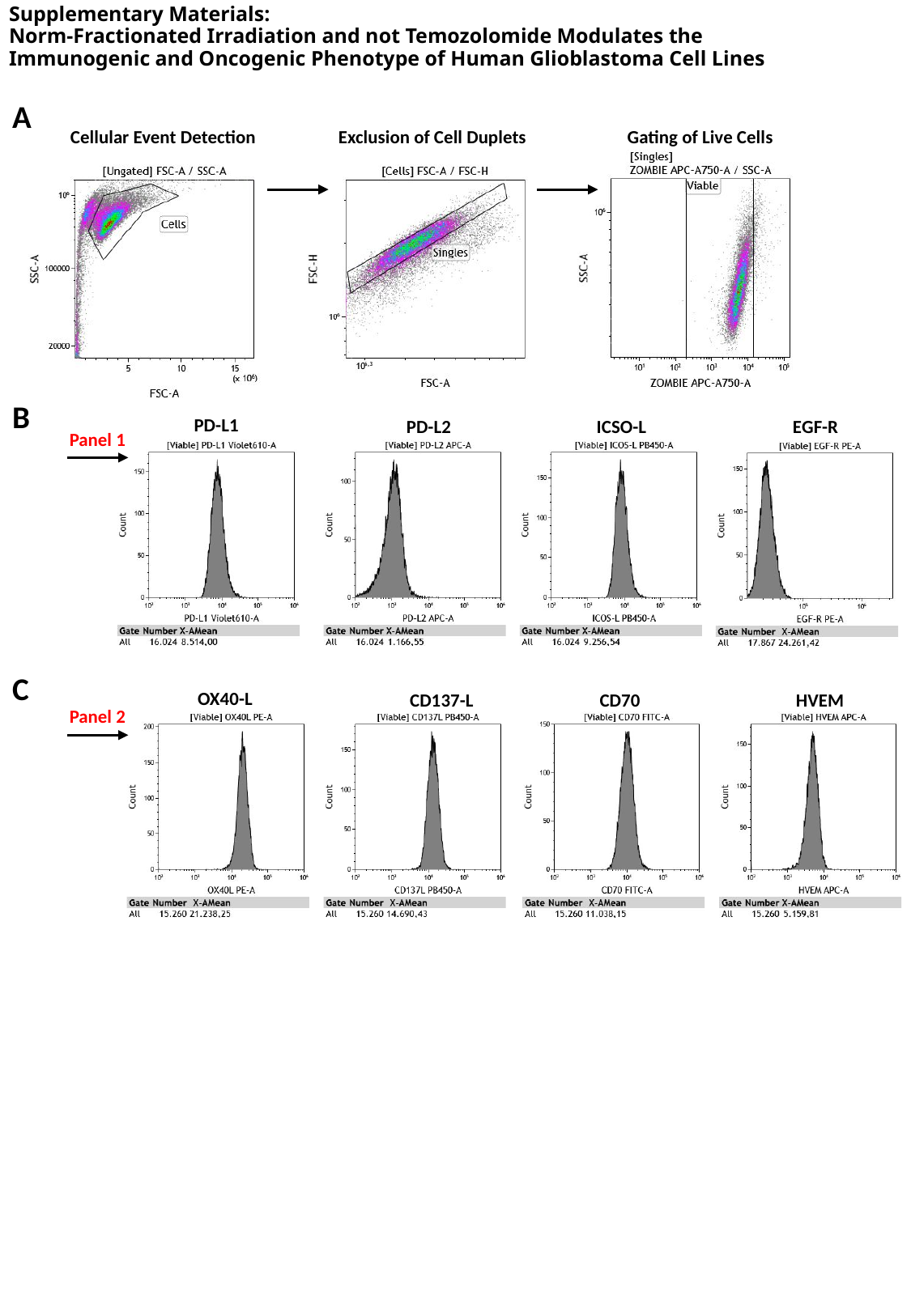

# Supplementary Materials: Norm-Fractionated Irradiation and not Temozolomide Modulates the Immunogenic and Oncogenic Phenotype of Human Glioblastoma Cell Lines
A
Cellular Event Detection
Exclusion of Cell Duplets
Gating of Live Cells
B
PD-L1
PD-L2
ICSO-L
EGF-R
Panel 1
C
OX40-L
CD137-L
CD70
HVEM
Panel 2
