# Supplementary material for: Normofractionated irradiation and not temozolomide modulates the immunogenic and oncogenic phenotype of human glioblastoma cell lines
Source: Strahlenther Onkol. 2022 Dec 8;199(12):1140–51. doi: 10.1007/s00066-022-02028-8 (PMC10673751; doi:10.1007/s00066-022-02028-8)
Supplement: Supplementary file 2 — Supplementary Fig. 2: Exemplary histogram of PD-L1 surface expression of mock-treated and irradiated U118 and U251 Cells. (A) U118 and (B) U251 cells were analyzed for cell surface expression of PD-L1 24 h after mock treatment or fractionated radiotherapy (RT) (5 × 2 Gy) for 5 consecutive days. Each experimental sample was analyzed in two test samples via flow cytometry, one with the stained panel (all antibodies plus the cell dye zombie included) and the other one unstained (only cell dye zombie included), to take the autofluorescence of the treated cells into account. Exemplary histograms show PD-L1 surface expression of U118 (A) and U251 (B) cells after mock treatment and irradiation. [file 66_2022_2028_MOESM2_ESM.pptx]

## Slide 1
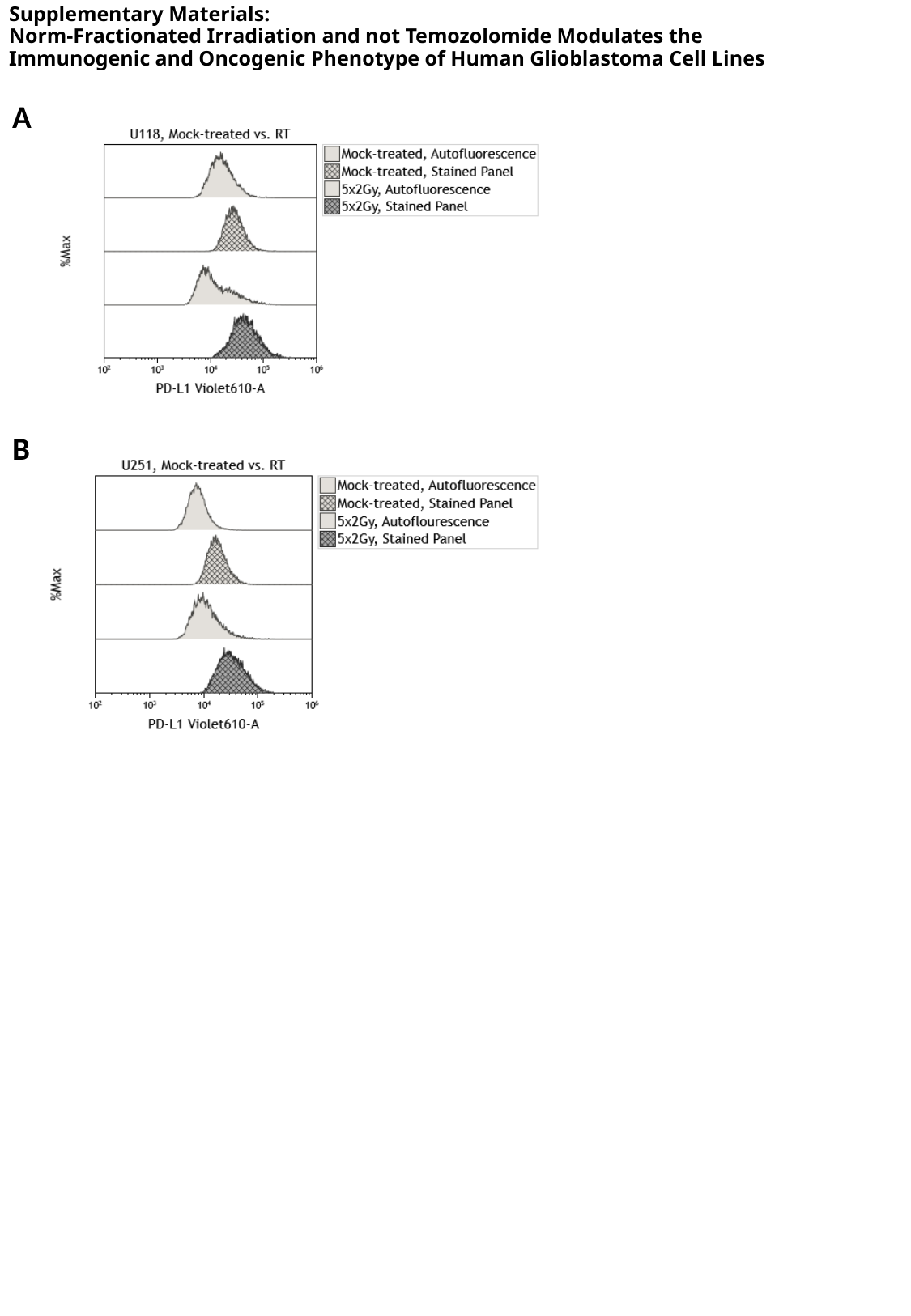

# Supplementary Materials: Norm-Fractionated Irradiation and not Temozolomide Modulates the Immunogenic and Oncogenic Phenotype of Human Glioblastoma Cell Lines
A
B
